# Decellularization of Porcine Carotid Arteries: From the Vessel to the High-Quality Scaffold in Five Hours

**DOI:** 10.3389/fbioe.2022.833244

**Published:** 2022-05-16

**Authors:** Maria Stefania Massaro, Petra Kochová, Richard Pálek, Jáchym Rosendorf, Lenka Červenková, Uta Dahmen, Václav Liška, Vladimíra Moulisová

**Affiliations:** ^1^ Biomedical Center, Faculty of Medicine in Pilsen, Charles University, Pilsen, Czechia; ^2^ New Technologies for Information Society-NTIS, University of West Bohemia, Pilsen, Czechia; ^3^ Department of Surgery, Faculty of Medicine in Pilsen, Charles University, Pilsen, Czechia; ^4^ Experimental Transplantation Surgery, Department of General, Visceral and Vascular Surgery, University Hospital Jena, Jena, Germany

**Keywords:** porcine carotid artery, optimized decellularization, scaffold quality, ECM proteins, endothelial cell adhesion, mechanical properties

## Abstract

The use of biologically derived vessels as small-diameter vascular grafts in vascular diseases is currently intensely studied. Vessel decellularization provides a biocompatible scaffold with very low immunogenicity that avoids immunosuppression after transplantation. Good scaffold preservation is important as it facilitates successful cell repopulation. In addition, mechanical characteristics have to be carefully evaluated when the graft is intended to be used as an artery due to the high pressures the vessel is subjected to. Here, we present a new and fast decellularization protocol for porcine carotid arteries, followed by investigation of the quality of obtained vessel scaffolds in terms of maintenance of important extracellular matrix components, mechanical resistance, and compatibility with human endothelial cells. Our results evidence that our decellularization protocol minimally alters both the presence of scaffold proteins and their mechanical behavior and human endothelial cells could adhere to the scaffold *in vitro*. We conclude that if a suitable protocol is used, a high-quality decellularized arterial scaffold of non-human origin can be promptly obtained, having a great potential to be recellularized and used as an arterial graft in transplantation medicine.

## 1 Introduction

Significant progress has been made in the last 20 years on the development of treatments to resolve small-diameter vascular diseases. Nevertheless, there is still lack of standardized procedures (Conklin et al., 2002; Chandra and Anthony, 2019; Seiffert et al., 2021). Autologous vessels, the currently most used solution, are difficult to harvest and can cause problems at the harvesting site that are not really desirable for the patient (Nagaoka et al., 2014). The use of artificial grafts, which are made of polytetrafluoroethylene (PTFE) or polyethylene terephthalate (such as Dacron), causes infection risks (Carrabba and Madeddu 2018). Furthermore, artificial grafts in the small caliber scale are more likely to develop thrombosis because the material is not completely compatible with blood circulation. Smaller diameters increase the occlusion possibilities derived from surface-mediated platelet activation. The ideal solution would be a small-caliber biological matrix that enhances endothelial cell adhesion and growth, with the decellularized vessel being a very promising candidate.

Decellularized tissues have already been approved and are currently used for clinical applications (Parmaksiz et al., 2016). The advantage of decellularized scaffolds lies in the removal of immunogenic cellular components that allows limiting or even avoiding immunosuppression (Massaro et al., 2021a). Moreover, well-decellularized tissues retain the major proteins and extracellular matrix components. These proteins are a real gain in biologically derived scaffolds because they enhance cell adhesion and growth, fundamental characteristics in tissue regeneration and healing (Schmidt and Baier 2000). Decellularized small-diameter blood vessels are still under development in terms of xenogeneic species of origin, decellularization protocols, and cell seeding. The selection of a suitable donor species and a vessel type has to be carefully considered in terms of dimensions and availability of the graft. Previous studies have focused on the use of porcine (Sheridan et al., 2014; Gu et al., 2018; Cheng et al., 2019; Cai et al., 2020), ovine (Li et al., 2016; Ilanlou et al., 2019), and canine (Cai et al., 2009; Zhou et al., 2009) carotid arteries with an inner diameter around 5 mm or smaller. The research has already led to a commercial product from decellularized bovine carotid arteries called Artegraft (North Brunswick, NJ), with very promising results from a 5-year follow-up study (Lindsey et al., 2018).

Despite this, there are still issues linked to the use of such material related to the immunologic reaction caused by inefficient decellularization (Chemla and Morsy 2009) and the lack of standardized decellularization procedures to obtain a good scaffold quality in reasonable time.

All available decellularization protocols require procedures lasting way more than 24 h, mostly even days (Dahl et al., 2003; Roy et al., 2005; Dahan et al., 2012; Sheridan et al., 2012; López-Ruiz et al., 2017; Wang et al., 2017; Gu et al., 2018; Cai et al., 2019; Cheng et al., 2019; Cai et al., 2020). Ideally, the decellularization protocol should be as fast as possible, while keeping the ultrastructure intact. Successful recellularization depends on good preservation of the individual scaffold components and their protein conformation (Gilpin and Yang 2017), which is, in turn, pivotal in the active functionalization of the tissue in terms of fast recreation of the endothelial cell layer, lowering the risk of thrombosis in the graft.

Considering our ongoing work on the decellularization of porcine liver (Moulisová et al., 2020) and porcine caval veins (Massaro et al., 2021b), our aim was to address these issues by developing a faster decellularization protocol for porcine carotid arteries with higher flexibility in obtaining the tissue scaffold and resulting in an ECM scaffold of high quality at the same time.

## 2 Materials and Methods

### 2.1 Experimental Design

**FIGURE 1:**
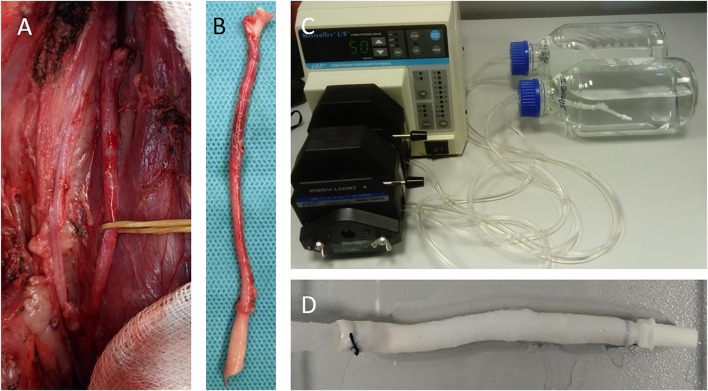
Explant and decellularization of carotid arteries: **(A)** carotid artery identified in the animal before the explant, **(B)** explanted carotid artery – the proximal side is at the bottom of the picture, and it is identifiable by its higher diameter if compared to the distal part that has the branches for the internal and external carotid arteries. The decellularization setup is shown in **(C)**; the masterflex pump speed is set to 50, but its corresponding value depends on the tube type and dimensions; and in our case, it corresponds to 250 ml/min. The decellularized artery scaffold is shown in **(D)**. The arteries are explanted from the animals and then rather decellularized within 24 h or stored for the long term at −80°C in a cryoprotective solution.

The initial prerequisite for preparing a good-quality vessel scaffold through decellularization is to prevent any damage to the source tissue that would decrease the preservation of the ECM. Thus, all porcine carotid arteries were harvested from anesthetized animals at our surgical facility to obtain fresh tissues (Figures 1A and B). The vessels were either subjected to decellularization within 24 h, or mechanical testing within 3 h, or cryopreservation for long-term storage and later processing. Characterization of scaffolds after decellularization was performed by testing mechanical properties, by assessing the level of decellularization, and by identification of important ECM proteins by histological and immunofluorescence techniques. Finally, the cytocompatibility of the scaffolds was analyzed by seeding endothelial cells and by visualizing cells by H&E staining and immunofluorescence.

### 2.2 Tissue Harvesting and Storage

The left and right carotid arteries (*n* = 46) were extracted from healthy Prestice black-spotted pigs (*n* = 23) aged 2–3 months and weighing 30–40 kg. The animals were premedicated by intramuscular administration of 10 mg/kg ketamine (Spofa, a.s., Czech Republic), 5 mg/kg azaperon (Janssen Pharmaceutica NV, Belgium), and 1 mg atropine (Hoechst Biotika, Slovak Republic). Propofol (1% mixture 5–10 mg/kg/h) was used for general anesthesia (Fresenius Kabi Norges as, Norway) and fentanyl (1–2 μg/kg/h) (Chiesi CZ s.r.o., Czech Republic) for analgesia. The animals were intubated and mechanically ventilated. Carotid explantation was initiated by making two longitudinal incisions on both sides of the neck of the pig parallel to the sternomastoid muscles. Common carotid arteries were gently dissected from the level of the brachiocephalic trunk until the branching into internal and external carotid arteries (Figure 1A). The arteries were then resected in the whole length (Figure 1B) and used either as fresh (fresh native – FN, fresh decellularized – FD) or cryopreserved (cryopreserved native – CN, cryopreserved decellularized – CD) and stored at −80°C for at least 2 months.

Native vessels (FN) were kept in saline on wet ice and tested for mechanical properties for up to 3 h. The vessels to be decellularized (FD) were stored in sterile saline at 4°C overnight (up to 16 h), prior to decellularization. The vessels to be decellularized after the freezing step (CD) were stored in Dulbecco’s modified Eagle medium (DMEM, Biosera Europe, France), supplemented with 10% fetal bovine serum (Biosera Europe, France), and 10% dimethyl sulfoxide (DMSO, Serva, Germany). Carotids tested as native after freezing (CN) were stored in saline supplemented with cryoprotective 10% DMSO. The samples to be cryopreserved were kept in Mr. Frosty™ Freezing Container (TermoFisher Scientific, MA, United States) at −80°C overnight, ensuring gradual cooling down (1°C/min), and then stored at −80°C for 2 months. The animals were euthanized under deep anesthesia with cardioplegic solution. All the procedures were performed under the approval MSMT-15629/2020-4 granted by the Ministry of Education, Youth and Sports of the Czech Republic, compliant with EU legislation. These animals were part of an approval for training of medical students in surgical techniques, and the carotid extraction took place immediately before euthanasia to maximize the use of animals in research in accordance with the 3Rs principle.

### 2.3 Decellularization Protocol

Freshly explanted vessels were stored in saline at 4°C and decellularized within 16 h, while cryopreserved vessels were stored for at least 2 months. At the time of decellularization, the vessels were thawed for 1 h at room temperature before starting the procedure. A 5-mm ring of the native artery from the proximal region was cut and fixed in 4% formaldehyde (Bamed s.r.o., Czech Republic) for histological comparison with the decellularized sample. The artery was then ligated at both ends to Luer connectors with a 3–0 Mersilk ligature (Ethicon Inc., NJ, United States) and connected to the decellularization setup consisting of flexible plastic tubing (Tygon S3, Saint Gobain, France). The two ends of the tubes were immersed in a glass bottle with a modified cap to allow the passage of the tubing. The tubing was inserted in a recirculating perfusion system driven by a peristaltic pump (Masterflex, Cole Parmer, IL, United States). The vessels were inserted while respecting the natural blood flow in the arteries from the proximal part toward the distal using a flow rate of 250 ml/min. The perfusion system is shown in Figure 1C, and the running saline cycle is documented in Supplementary Video 1. The first perfusion step was a saline wash for 10 min to clear the vessel of eventual blood clots and of the residues of cryoprotective solution in case of cryopreserved samples. Then, two successive perfusion cycles with 1% Triton X-100 solution for 1 h were applied, followed by two cycles of 1 h of 1% sodium dodecyl sulfate (SDS) solution. Both the detergent solutions were prepared by dissolving Triton X-100 (Carl Roth, Germany) and SDS pellets (Carl Roth, Germany) in sterile ultrapure deionized water (resulting pH for Triton X-100 and SDS solutions was 6 and 7, resp.). Finally, four consecutive washing steps lasting 15 min using saline were used to remove residual SDS from the scaffold. At the end of the procedure, the decellularized vessel was removed from the system, a 5-mm segment was harvested from the proximal region for histological evaluation, and the vessel was kept in a storing solution (PBS complemented with 1% penicillin/streptomycin and 0.2% amphotericin B (Cat. Nos. 15140122 and 15290018, respectively, Gibco, Paisley, United Kingdom)) at 4°C until further use. All the procedures were conducted under sterile conditions. For the *in vitro* assays, additional washing steps were applied to the scaffolds: The scaffold was added in a container with 100 ml of new solution used for storage and agitated for 24 h at room temperature. Then, the solution was changed, and the same wash was repeated to obtain a 2-day washed scaffold. By repeating this washing, a 4-day washed scaffold was prepared.

### 2.4 Histology and Immunofluorescence

Proximal parts of the arteries were fixed in neutral-buffered formalin (Bamed, Czech Republic), dehydrated in ethanol, and embedded in paraffin blocks. The tissue was cut to obtain 5-µm sections using a microtome (Leica RM2255, Germany). The sections were deparaffinized, rehydrated, and stained using six histological methods and four immunofluorescence (IF) stainings as described hereafter.

*Hematoxylin and eosin (H&E) staining* was used to assess the efficiency of decellularization in terms of the remaining nuclei in scaffolds decellularized from fresh tissue (freshly decellularized, FD, *n* = 12) or after cryopreservation (cryopreserved decellularized, CD, *n* = 17). The number of samples differs between the two groups because while fresh samples were used only in mechanical testing, the cryopreserved arteries were used for both mechanical testing and endothelial cell seeding. A scoring system from 0 to 3 was introduced to quantify the decellularization efficacy; score 0 refers to complete decellularization, while score 3 means that less than 50% of the section area was decellularized (details in Supplementary Table 1). Whole slide images were processed in this analysis, and scans were generated on Zeiss Axioscan.

*Verhoeff’s hematoxylin with green trichrome staining* (Kocová 1970) and *orcein staining* (Tanzer’s orcein, Bowley Biochemical Inc., Danvers, MA, United States) were used to stain elastic fibers. *Picrosirius red staining* (Direct Red 80, Sigma-Aldrich, Germany) was used for visualization of collagen fibers in polarized light (Lattouf et al., 2014). *Standard Alcian blue staining* (pH 2.5) combined with periodic acid–Schiff (PAS) was used for the detection of both neutral mucins and acidic glycosaminoglycans (GAGs).

Immunofluorescence staining of paraffin sections was used to further characterize the completely decellularized matrix. Primary antibodies for *collagen I* (1:400, MA1-26771, Invitrogen, MA, United States), *elastin* (1:100; ab21610, Abcam, United Kingdom), *fibronectin* (1:200, ab6328, Abcam, United Kingdom), and *laminin* (1:30, L9393, Sigma-Aldrich, Germany) were selected. Goat anti-mouse AF 568 (1:200, A11004, Invitrogen, MA, United States) was used as a secondary antibody for collagen I and fibronectin, while chicken anti-rabbit AF 647 (1:200, A21443, Invitrogen, MA, United States) was used as a secondary antibody for elastin and laminin. For IF staining of samples from *in vitro* experiments, the whole formaldehyde-fixed vessel pieces were used (see Section 2.7). Cellular actin was detected using CruzFluor 488 phalloidin (1:1,000, Santa Cruz Biotechnologies, TX, United States), the cell nuclei were stained with 1 μM DAPI solution (Sigma-Aldrich, Germany), and the scaffold was visualized by staining with collagen I antibody in the same dilution as for the sections mentioned earlier. For imaging the sample surface with cells, the vessels were flattened on the microscopic slide using a coverslip. All IF images were obtained using a fluorescence microscope Olympus IX80 either at 10x or 20x magnification.

### 2.5 Mechanical Testing

To evaluate the effect of the decellularization procedure on the mechanical strength of the scaffold, the native non-decellularized vessels were compared with the decellularized arteries. To further characterize the scaffold, both fresh and cryopreserved tissues were tested. Four different groups of arteries were used: freshly explanted (fresh native, FN, *n* = 10), frozen native (cryopreserved native, CN, *n* = 8), decellularized from fresh tissue (fresh decellularized, FD, *n* = 8), and decellularized from frozen tissue (cryopreserved decellularized, CD, *n* = 6); the storage conditions are detailed in Section 2.1. The number of samples in each group was different due to processing and decellularization scores (only scaffolds with scores 0 and 1 were used). The FN samples were kept at 4°C for storage/transport and used within 3 h from the explant. The CN specimens were thawed in a water bath at 37°C for 10 min to reduce the damage due to potential recrystallization inside the cells (Amini et al., 2020). The arteries were allowed to assimilate to room temperature for 20 min before starting the mechanical measurement. Each vessel (total length was about 100 mm) was divided into three sections: distal, middle, and proximal. A detailed photo-documentation (Olympus camera) and measurement of the size and shape of the whole artery, artery segments, diameters, and rings were carried out. Larger specimens were pictured next to the ruler (Supplementary Figure 1A), whereas smaller artery segments and arterial rings were placed on graph paper (Supplementary Figure 1B). Pictures of arterial rings from the edges were used to directly assess the initial cross-sectional area of the artery using Ellipse software (ViDiTo Inc., Slovak Republic). Briefly, the picture was calibrated according to the graph paper (a square is equivalent to 1 × 1 mm), then the outer and inner diameters were manually annotated, followed by the calculation of circular areas (Supplementary Figure 1C). Finally, the initial artery cross-sectional area was calculated as the average of the difference between the area of the outer and inner circle of artery edges.

#### 2.5.1 Determination of Opening Angle

After measuring the cross-sectional area, the rings were opened, and the opening angle was measured after 15 min incubation in saline. The opening angle was defined as the angle formed between two lines starting from the middle point of the inner layer and passing from the middle part of the wall in the opened region, as shown in the scheme in Figure 6C.

#### 2.5.2 Determination of the Uniaxial Tensile Properties

For uniaxial mechanical tension tests, each sample was clamped with the jaws of the mechanical measurement device (574LE2, TestResources Inc., MN, United States). Ten cycles of stretching to 15% of the initial length were performed to precondition the arteries before the actual measurement was started. The test consisted of pulling on both ends. The initial length of the sample inside the jaws was 10 mm. Then, the samples were loaded with a linear increasing elongation until the tissue ruptured at 6 mm/min, which corresponds to 1% per second. The initial cross-sectional area for each individual segment and initial length was used to estimate the stress and strain of the samples. The stress (*σ*) was simply calculated as the ratio between the force detected by the load cell on the sample (*F*) and the initial cross-sectional area (*A*_*0*_). The strain (*ε*) was the actual elongation (*l*) divided by the initial length value (*l*_*0*_). Young’s modulus (*E*) was calculated as the ratio between the stress and strain.

$$\sigma =\frac{F}{A_{0}}\left[MPa\right]\;\;\;\;\;\;\;\;\;\;\;\;\varepsilon =\frac{l-l_{0}}{l_{0}}\;\;\;\;\;\;\;\;\;\;\;\;\;\;E=\frac{\sigma }{\varepsilon }\left[MPa\right].$$

Given that in the stress–strain curve we can identify two main regions for soft tissues, Young’s modulus is divided into E_0_ and E_1,_ which represent the elastic and the viscoelastic behavior of the sample, respectively. These regions correspond to a linear increase in the stress–strain curve.

### 2.6 SDS Residue Assay

The SDS amount in scaffold washing solutions was quantified using the Residual SDS Detection Kit (BioBasic Inc., Canada), following the manufacturer’s protocol. Briefly, undiluted samples were mixed with solutions A and B, then vortexed for 30 s, left standing for 5 min at room temperature, and then centrifuged at 5,000 rpm for 5 min. The optical density of the sample supernatants and that of the standard solutions was measured at 499 nm using a Synergy HT microplate reader (Biotek, CA, United States). The final SDS concentration in the samples was obtained through a linear interpolation of the calibration curve derived from the optical density of standard SDS solutions. Each sample condition (after decellularization, 2-day wash, and 4-day wash) was an average of four different experiments, and the samples were tested in duplicates.

### 2.7 *In Vitro* Experiments

Human umbilical vein endothelial cells (HUVECs, PromoCell GmbH, Germany) and rat aorta endothelial cells (RAECs, obtained from the laboratory of Prof. Dahmen, Universitatklinikum Jena) were cultivated in Endothelial Cell Growth Medium (C-22010, PromoCell GmbH, Germany) and used at passages not higher than seven. Decellularized arteries after 2-day or 4-day washing (described in Section 2.3) were cut in half to expose the inner layer and then cut into smaller pieces (about 1 cm long). The scaffold pieces were placed in a 48-well plate, and 500 μl of cell suspension was dropped on the top of each sample at a seeding density of 10,000 cells/cm^2^. The vessels were incubated in a humidified incubator at 37°C and 5% CO_2_ for 24 h. Then, the samples were processed to assess cell viability using a Live/Dead Viability/Cytotoxicity kit (Invitrogen, L3224, MA, United States) according to the manufacturer’s manual. For histochemical and immunofluorescence staining, the sample replicates were washed in phosphate-buffered saline (PBS), fixed in 4% formaldehyde (Bamed s.r.o., Czech Republic), and either embedded in paraffin for H&E staining (5 μm sections) or used as the whole sample for immunofluorescence staining for visualization of collagen I from the scaffold and cell cytoskeleton and DNA, as detailed in Section 2.4.

### 2.8 Statistical Analysis

The normality of individual data sets was tested using the Shapiro–Wilk’s *W* test. Data sets with a normal distribution of values were analyzed using a two-way ANOVA test with Tukey’s multiple comparison post-test. Data sets which did not pass the test for normality were analyzed using the Kruskal–Wallis test with Dunn’s post-test to compare individual groups. GraphPad Prism 7.0 software (GraphPad Software Inc., CA, United States) was used for all statistical analyses. The decellularization score was analyzed using the Mann–Whitney test. The *p*-value representing a statistically significant difference was set at <0.05.

## 3. Results

### 3.1 Decellularization

The decellularization was always performed by processing two vessels at the same time. The total time for achieving complete decellularization was 5 h and 10 min, with all the vessels turning white at this point (Figure 1D). Nevertheless, histological staining with H&E revealed that there were differences in the efficiency of decellularization. In some cases, the scaffold was completely decellularized, while in others, a specific area of the tunica media still contained nuclei. We introduced a scoring system based on the percentage of nuclei in the entire circular wall section. The score was set from 0 for completely decellularized up to 3 for more than 50% of the area still containing nuclei; for details, see Supplementary Table 1. Interestingly, arteries that were decellularized from the fresh tissue (FD) scored better in terms of complete or nearly complete decellularization (scores mainly 0 and 1), while vessels decellularized from the frozen tissue (CD) did not score that well (scores ranged between 0 – 3 with the majority of the values being 1 and 2). Statistical analysis revealed that this difference between CA decellularization protocols (fresh or frozen tissue used for decellularization) was significant, favoring the use of fresh tissue (Supplementary Figure 2C).

**FIGURE 2:**
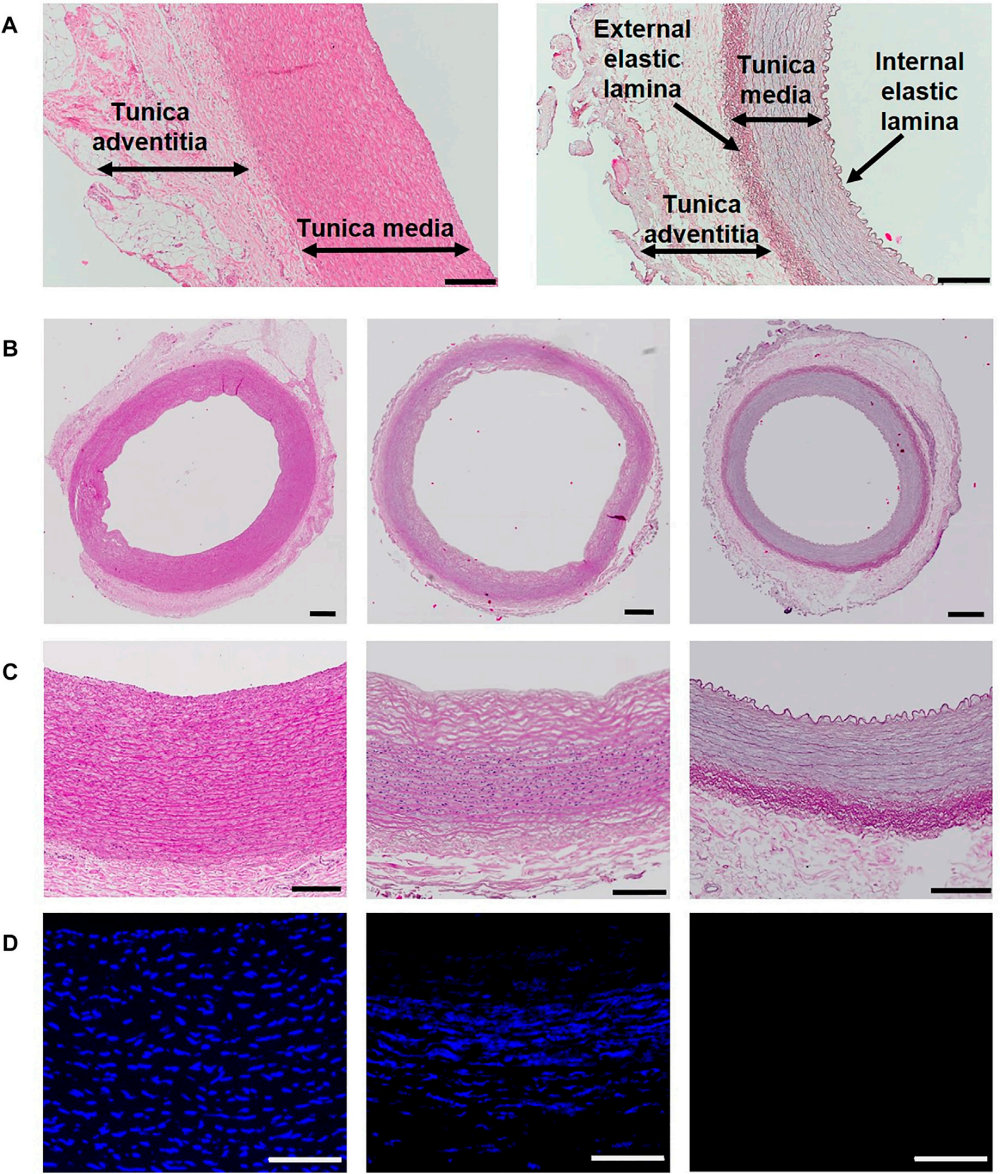
Porcine coronary artery layers and evaluation of the decellularization efficacy: **(A)** detailed image of the native (left) and decellularized (right) carotid artery wall with the identification of the wall layers. It is evident that in the native vessel, the internal and external elastic laminas between each layer are indistinguishable, while they can be easily identified in the decellularized scaffold. The scale bar is 200 μm. **(B)** H&E staining of the entire section (native on the left, partially decellularized (score 2) in the middle and completely decellularized scaffold on the right) – scale bar is 500 μm; **(C)** details of the vessel wall from pictures in **(B)** to visualize the nuclei stained with hematoxylin – scale bar is 200 μm; **(D)** DAPI immunofluorescence at magnification ×40 showing nuclear loss in the middle picture and complete cell removal on the right side) scale bar is 100 μm.

H&E and DAPI staining allowed us to visualize the nuclei and protein content in the native and decellularized carotids (Figure 2A). The variability among samples related to the different levels of decellularization is displayed in Figures 2B,C. There, the H&E staining shows the native artery section (left), a partially decellularized vessel that scored 2 (middle), and a completely decellularized scaffold scoring 0 (right). The purple-stained nuclei from H&E images corresponded with the DAPI-stained nuclei (Figure 2D). An overview of the CD and FD vessel scaffolds is given in Supplementary Figures 2A and B, respectively, highlighting the general morphological variability among animals.

**FIGURE 3:**
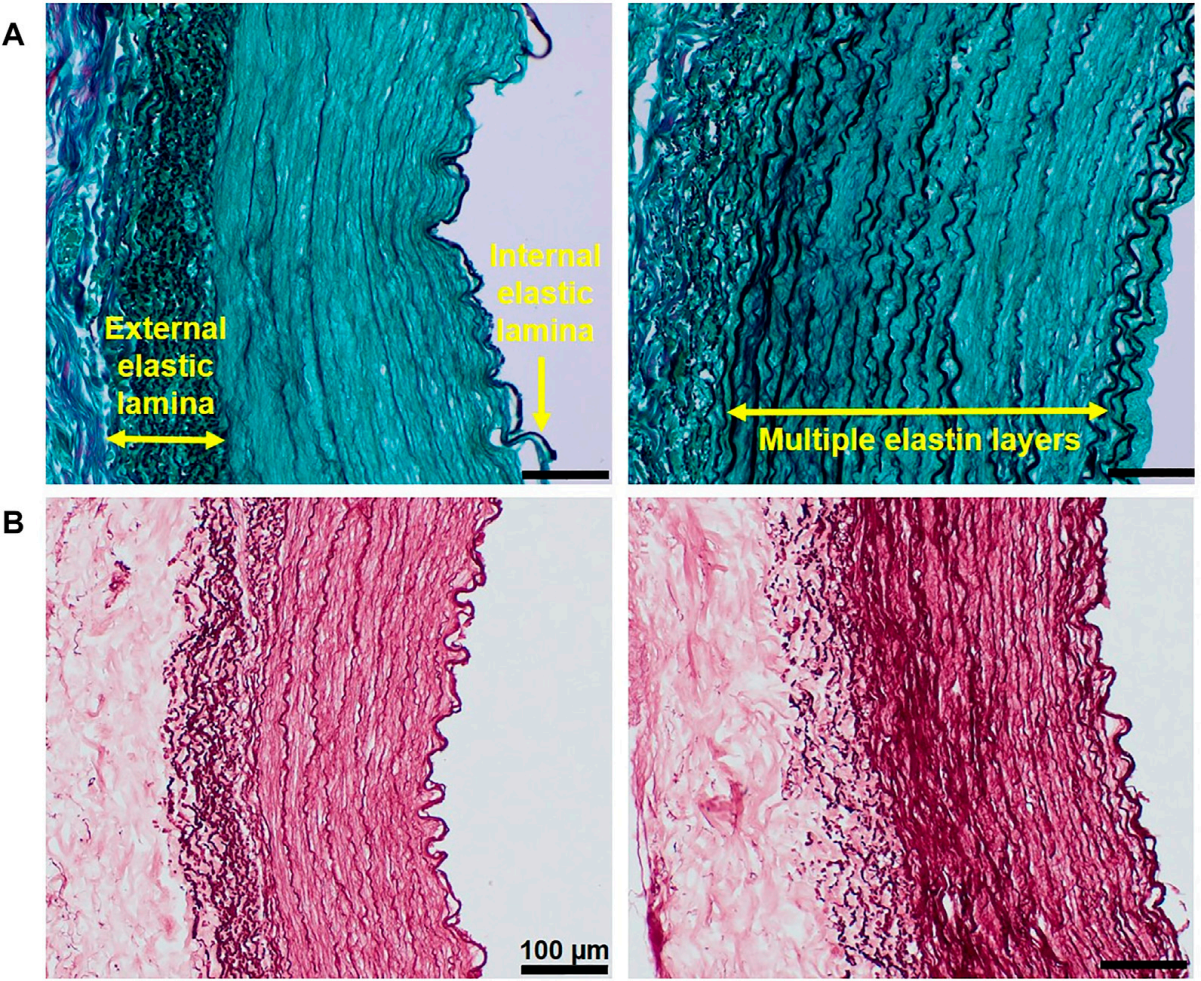
Visualization of the highly variable presence of elastic fibers in decellularized arteries: Black color in Verhoeff’s green trichrome–stained samples **(A)** and dark red color in orcein-stained samples **(B)** represent elastic fibers which are always present in external and internal elastic lamina; however, the amount of these fibers in tunica media is much lower in the sample on the left-hand side (weaker staining of elastic layers) than in the multiple elastic layers strongly stained in the sample on the right-hand side. The scale is the same for all the figures (100 µm).

The black color in Verhoeff’s green trichrome staining evidenced the highly variable elastin content in decellularized samples (Figure 3A) and in native vessels. The elastin content of individual decellularized vessels corresponded to their native counterparts, meaning the decellularization protocol does not affect the presence of this protein. In contrast, it demonstrated the preservation of these fibers. We observed that elastic fibers were always present in the external elastic lamina and internal elastic lamina, while tunica media showed different amounts of elastic layers. Some samples were stained rather weakly (Figure 3, left column), while others showed strong staining of these multiple layers in parallel orientation (Figure 3, right column). To confirm the elastin presence, orcein staining was used to show elastin fibers in dark red (Figure 3B).

**FIGURE 4:**
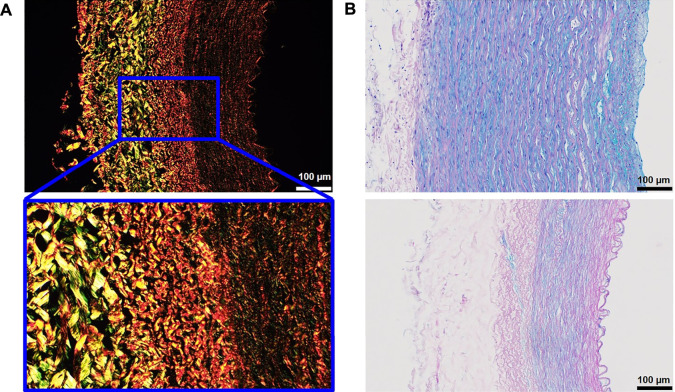
Collagen and glycosaminoglycans (GAGs) in decellularized arteries. **(A)** Collagen fibers visualized by Picrosirius red staining in polarized light. **(B)** Alcian blue staining in native (top) and decellularized (bottom) tissue shows the presence of GAGs; the loss of blue staining in decellularized tissue can be caused by the removal of GAGs present within cells.

Collagen fibers were also mostly intact after the decellularization, as shown not only by pink color staining from H&E images (Figures 2B and C) but also by light green color in Verhoeff’s green trichrome staining (Figure 3). Moreover, we used Picrosirius red staining imaged in polarized light to visualize birefringent collagen molecules and reveal the presence of different collagen types in individual layers of the carotid artery (Figure 4A). The adventitia and external elastic lamina showed a strong yellow-red signal associated with collagen I, while tunica media had weaker staining with greenish color linked to collagen III (Junqueira et al., 1979).

In order to detect the presence of glycosaminoglycans (GAGs), which are important for non-structural functions of the ECM such as binding of various cytokines and growth factors (Gandhi and Mancera 2008), we used Alcian blue staining. The top image in Figure 4B shows a strong blue GAG staining in the tunica media of the native artery, whereas the adventitia contains almost no GAGs. The bottom image represents a typical example of a decellularized vessel and shows weaker GAG staining in the muscular tunica media in comparison to the native vessel. The loss of blue-stained GAGs after the decellularization can be associated with the removal of cells that have some GAGs bound on the membrane. We also cannot exclude that the detergents, particularly the SDS used for decellularization, could contribute to the reduction in GAG content in the ECM. Nevertheless, the level of blue staining in the decellularized vessel is still satisfactory to conclude that we have retention of some GAGs in the scaffold after decellularization.

**FIGURE 5:**
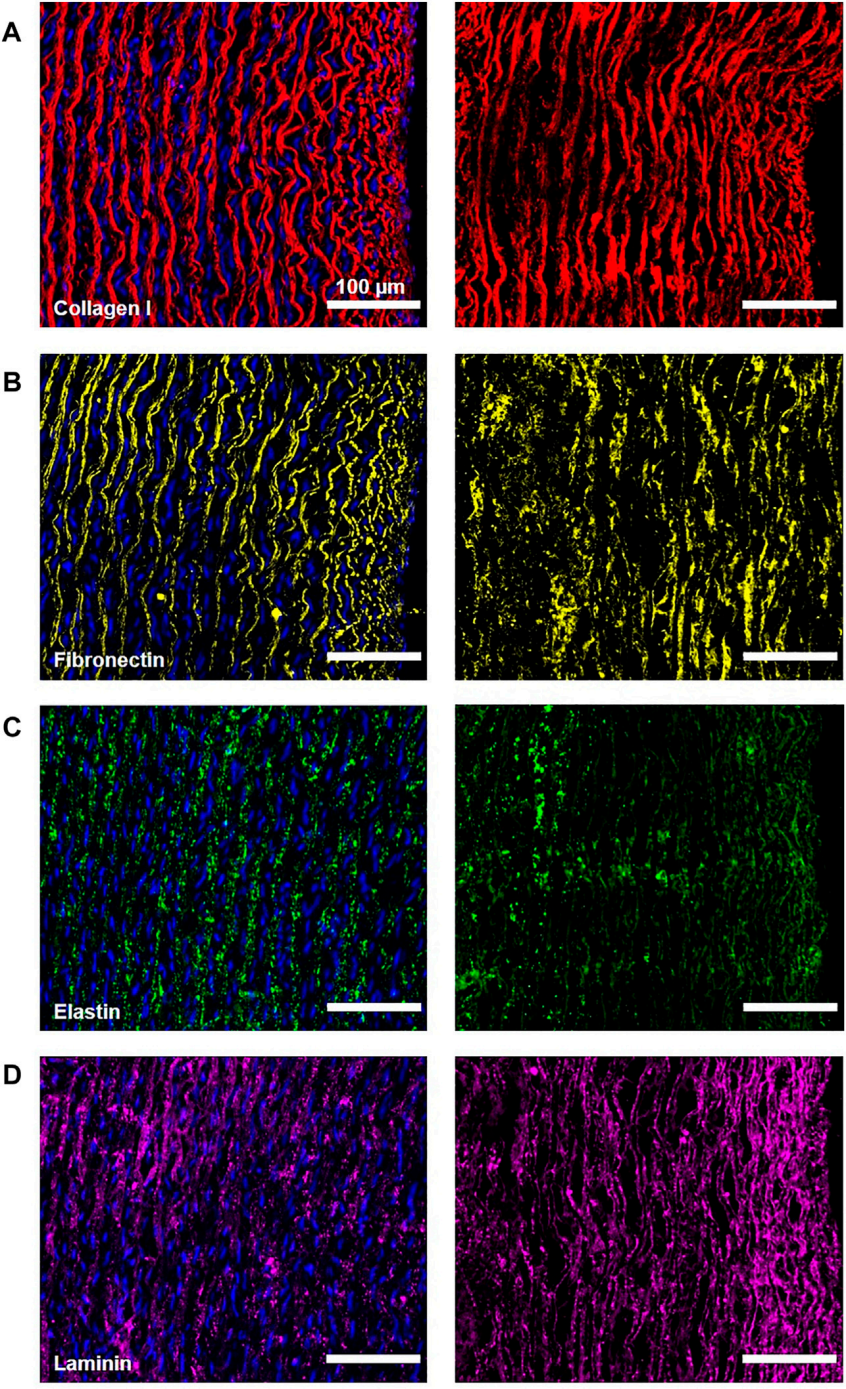
ECM proteins in carotid arteries before (left) and after (right) the decellularization visualized by immunofluorescence. The vessel intima is always oriented on the right-hand side of the image. Collagen I is stained in red **(A)**, fibronectin in yellow **(B)**, elastin in green **(C)**, and laminin in magenta **(D)** (all artificially assigned colors). Blue DAPI shows nuclei in the native artery, while absence in decellularized tissue is indicative of complete cell removal. The scale bar is 100 μm for all the images.

To visualize specific ECM proteins such as collagen I, fibronectin, elastin, and laminin, we performed immunofluorescence staining. All four proteins were shown to withstand the decellularization procedure, as evidenced in the representative images of native and decellularized tissues in Figures 5A-D. Although collagen I is also stained by some of the abovementioned histological techniques, we still performed the extra IF staining for this protein. Histochemical collagen staining signals give more general information regarding the presence or absence of collagen but do not differentiate between the subtypes of collagen. In contrast, immunofluorescence visualizes individual specific collagen subtypes.

We also characterized the scaffold’s ability to clear from detergent residues by SDS content assessment at different stages of washing. It is known that SDS detergent residues are cytotoxic for cells (Gilpin et al., 2014; Welch et al., 2021), with endothelial cells being particularly susceptible (Zvarova et al., 2016). As ECs are crucial for vessel repopulation, we analyzed the SDS concentration at the end of the decellularization procedure and after the application of several washing steps with PBS. As this scaffold characterization has a direct impact on cell culture, we show the SDS concentration data together with the *in vitro* results in Section 3.3.

### 3.2 Mechanical Testing

**FIGURE 6:**
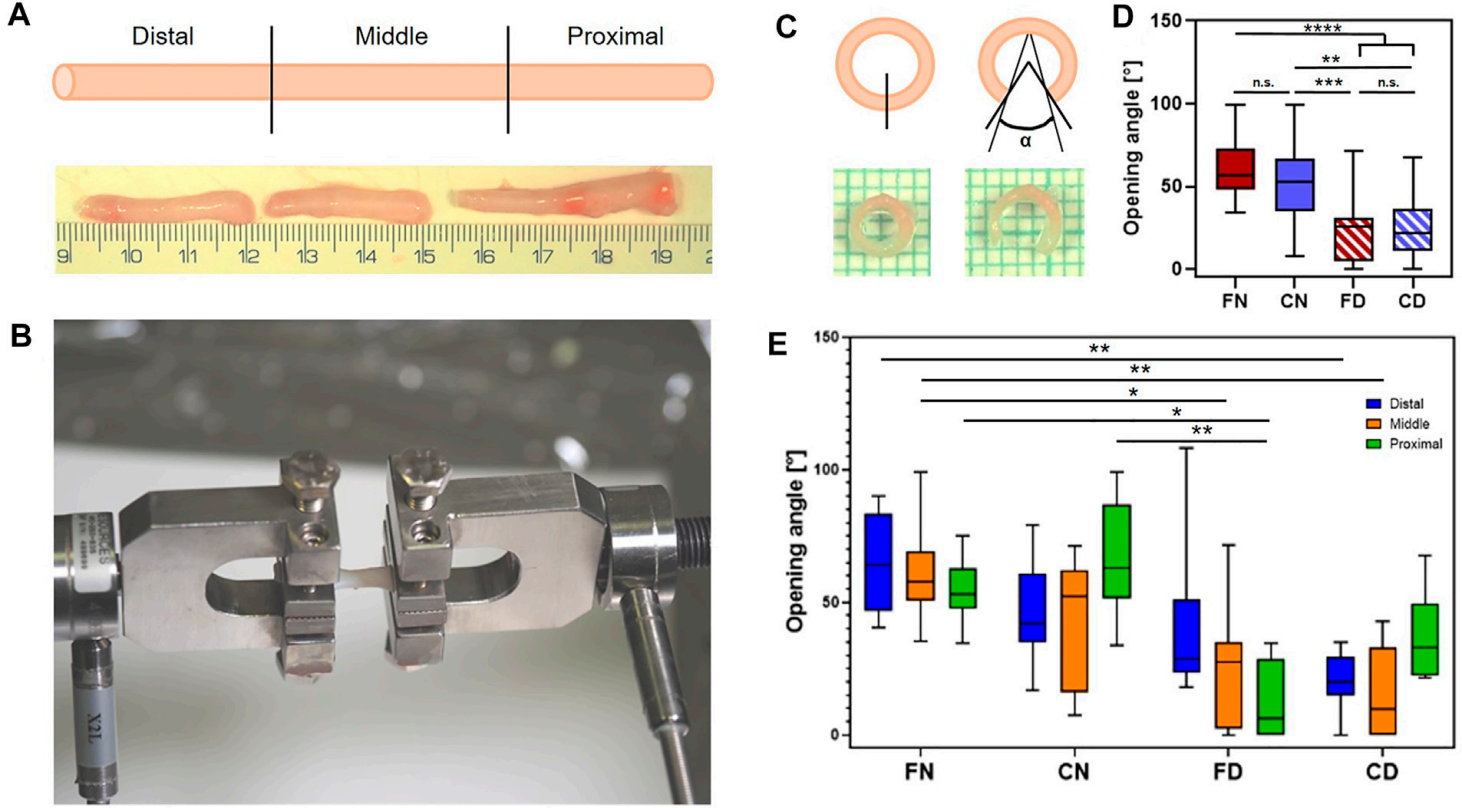
Uniaxial tensile mechanical testing of native and decellularized vessels: **(A)** Scheme of the three sections of the artery (top) and image of a native artery after the division (bottom). **(B)** Image of the sample at the start of the load application (initial length is 10 mm); **(C)** scheme of the opening angle measurement (top) and image of the sample before (bottom left) and after (bottom right) incision; **(D)** boxplot of the opening angle for the four groups of fresh/frozen native artery or decellularized scaffold; **(E)** boxplot of the opening angle with a comparison between individual segments and the four groups. Abbreviations: FN, fresh native artery; CN, cryopreserved native artery; FD, freshly decellularized scaffold; CD, cryopreserved decellularized scaffold; level of significance is displayed as (*) for *p* < 0.05, (**) for *p* < 0.01, (***) for *p* < 0.001, and (****) for *p* < 0,0001.

Maintaining the original mechanical properties is extremely important in the case of arterial grafts. Therefore, we paid special attention to the mechanical testing of the vessel scaffolds. We compared the mechanical properties of native arteries with the decellularized ones. We tested both vessels and scaffolds processed either as fresh tissue or as thawed tissue after cryopreservation. Considering the different levels of decellularization of the vessels in this study, only fully decellularized or nearly fully decellularized scaffolds were analyzed (scores 0 and 1). Vessels scoring 2 and 3 were discarded from this analysis as the mechanical properties might be affected by the remaining cellular material. As shown by previous studies (Kochová et al., 2012; Tomášek et al., 2020), morphometric parameters and mechanical properties can significantly vary in different segments of the carotid artery; thus, we divided each vessel into three parts, namely, distal, middle, and proximal segments (Figure 6A). These segments were then tested and analyzed separately. In order to compare our results with other studies that did not consider variations between segments, we also analyzed the data obtained from individual arteries by merging the data from all three segments. Supplementary Table 2 shows detailed values obtained during this analysis.

#### 3.2.1 Determination of the Opening Angle

First, the opening angle test was performed for each vessel segment. A scheme of the experiment is shown in Figure 6C. As expected, the opening angle decreased with the decellularization procedure. Fresh native (FN) vessels and cryopreserved native (CN) arteries showed significantly larger opening angles than the freshly decellularized (FD) and cryopreserved decellularized (CD) tissues (Figure 6D). The opening angles varied neither between the native tissues (FN and CN) nor between the decellularized scaffolds (FD and CD). The mean angles were 60° ± 16 and 52° ± 23 for the native groups and 22° ± 18 and 24° ± 18 for the decellularized groups. Due to the data not passing the normality test, a nonparametric Kruskal–Wallis method with Dunn’s post-test was used for statistical analysis.

Figure 6E shows the opening angle values for individual segments. The opening angle did not vary significantly between different regions (distal, middle, and proximal) within the same tested group. Values of 65° ± 19, 61° ± 17, and 55° ± 12 were obtained for FN segments in the three regions, respectively. In the same way, 46° ± 19, 44° ± 24, and 66° ± 22 were found for FS, 26° ± 5, 25° ± 23, and 12° ± 16 for FD, and 20° ± 12, 15° ± 17, and 37° ± 18 for SD. This set of data passed the normality test; thus, a two-way ANOVA test was used for statistical evaluation (using Tukey’s multiple comparison post-test). These results from segment analysis were in agreement with the more general analysis of the opening angle values when all segments were analyzed together. The nonsignificant opening angles of the corresponding segments (native vs. decellularized) were most probably caused by the smaller number of individual measurements when analyzing the segments separately. Overall, the opening angle of tested tissues was not dependent on the individual region but rather on the analyzed group.

#### 3.2.2 Determination of the Uniaxial Tensile Properties

**FIGURE 7:**
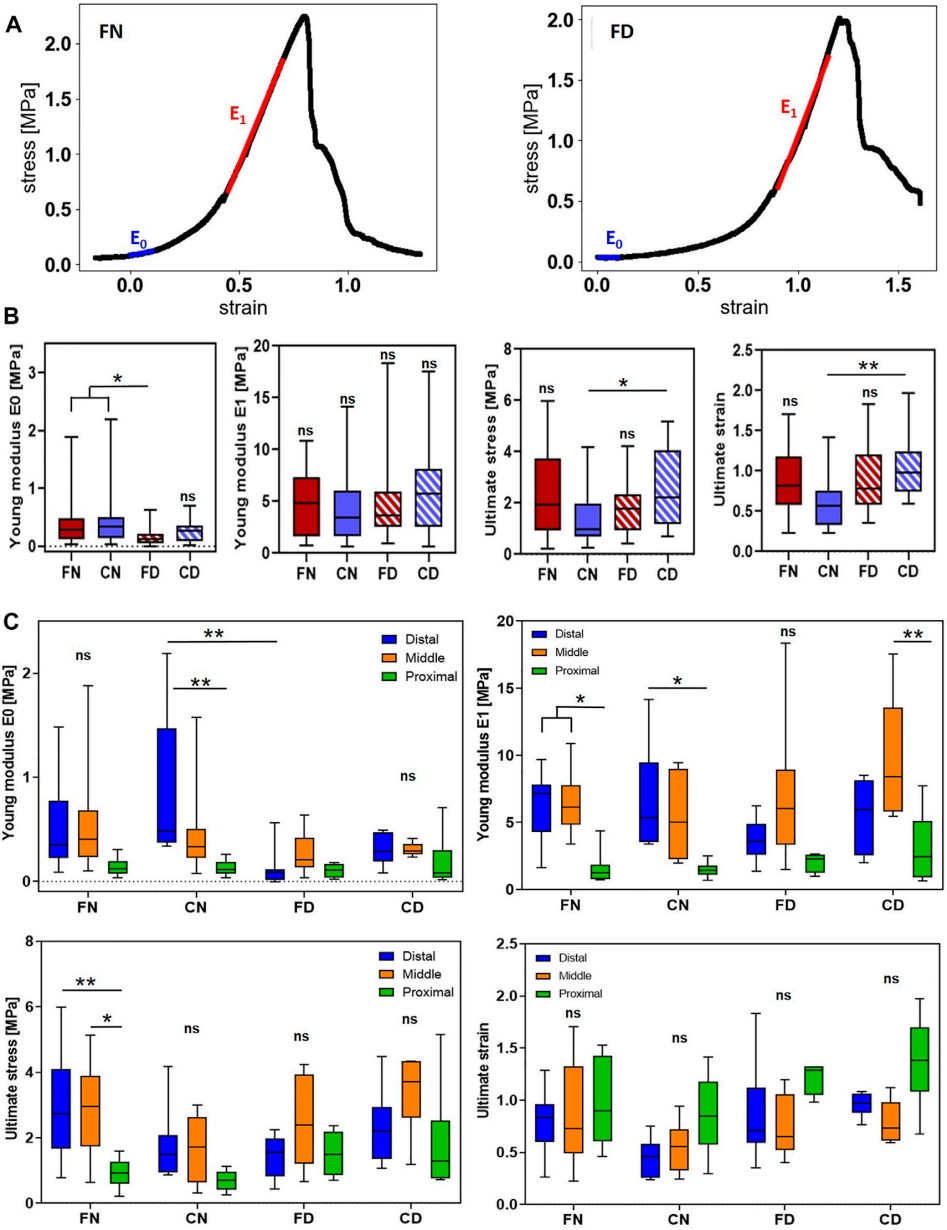
Mechanical testing: **(A)** Typical stress–strain curves for the distal region of a native carotid (left) and a decellularized scaffold (right) in both curves. Young’s modulus of elastic phase (E0) and the viscoelastic phase E1 and the rupture point (the highest value of stress reached) were easily identified. Boxplots of the mechanical test parameters are shown in **(B)** and **(C)**, where **(B)** represents parameters for the four main groups together, while **(C)** shows more detailed data of individual segments within the groups. FN, fresh native artery; CN, cryopreserved native artery; FD, freshly decellularized scaffold; CD, cryopreserved decellularized scaffold; level of significance is displayed as (*) for *p* < 0.05 and (**) for *p* < 0.01.

Uniaxial tensile mechanical testing with the procedure depicted in Figure 6B showed a nonlinear behavior of the arteries as typical for biological tissues (Kubíková et al., 2017; Kochová et al., 2019). Representative examples of the stress–strain curves are given in Figure 7A, and examples for all the four groups divided between proximal, middle, and distal sections are shown in Supplementary Figure 3. In these curves, two linear regions can be identified for all samples: the small deformation region (elastic phase dominated by elastin, highlighted in blue) and the large deformation region (viscoelastic phase dominated by collagen, highlighted in red). While in the elastic phase, the material retains its initial properties, which can be restored if the load is removed; in the viscoelastic phase, the properties of the materials start to change until the ultimate stress is reached, and the sample is broken at the rupture point. For every sample, we obtained the following individual mechanical parameters: Young’s modulus for both elastic phase (E_0_) and viscoelastic phase (E_1_) and ultimate stress and ultimate strain. These data were again analyzed for both the entire artery (Figure 7B) and three segments individually (Figure 7C).

##### 3.2.2.1 Comparison of the Whole Vessel

In the general analysis of whole vessels, the datasets for Young’s moduli E_0_ and E_1_ and for the ultimate stress did not have the normal Gaussian distribution; thus, the Kruskal–Wallis test was used for statistical evaluation. The ultimate strain dataset showed normal distribution and was analyzed by one-way ANOVA.

Young’s modulus of the elastic phase (E_0_) was significantly lower for scaffolds decellularized from fresh tissue (0.177 ± 0.181 MPa for FD and 0.264 ± 0.179 MPa for CD) than both groups of native arteries (0.403 ± 0.421 MPa for FN and 0.483 ± 0.556 MPa for CN), as visible in Figure 7B far left. This result suggests that some elastic components of the ECM might have been damaged during the decellularization procedure in this type of scaffold. However, this was not the case in scaffolds obtained from cryopreserved vessels. These scaffolds did not vary significantly from the native vessels, pointing out the good preservation of the elastic part of the ECM structure.

The values of Young’s modulus of the viscoelastic phase (E_1_) did not vary significantly between any sample groups, suggesting that the collagen structural components were not affected by the decellularization procedure (Figure 7B, middle-left). The values of 4.77 ± 3 MPa, 4.45 ± 3.55 MPa, 4.73 ± 3.93 MPa, and 6.09 ± 4.27 MPa were found for FN, CN, FD, and CD, respectively. The ultimate stress and ultimate strain resulted in similar values among the groups, with the only significant difference between cryopreserved samples CN and CD (with values of ultimate stress 1.38 ± 0.96 MPa for CN and 2.51 ± 1.46 MPa for CD and of ultimate strain 0.62 ± 0.32 for CN and 1.04 ± 0.37 for CD), as shown in Figure 7B middle-right and far-right. In these two cases, the values of ultimate stress and strain were surprisingly higher in decellularized samples than their native counterparts. The fact that the scaffold generated from cryopreserved tissue has slightly better mechanical properties than the native tissue that underwent a freeze–thaw cycle can be explained by a certain delay in the processing of the native vessel for the mechanical testing after thawing. The ECM of the CN vessel might be slightly damaged by enzymes released from the dead cells during the testing. The thawed vessels to be decellularized had always a shorter period of time between thawing and cell removal, leading to better preservation of the ECM. The ultimate stress and strain values of CD scaffolds actually reach the values identified for the fresh vessels (FN), confirming a good quality of the scaffold being generated from the cryopreserved vessels.

##### 3.2.2.2 Comparison of Specific Arterial Segments Among the Groups

The detailed analysis of the four mechanical parameters for individual arterial segments is shown in Figure 7C. Even with a reduced number of experimental data per condition, if compared with the evaluation of the four main groups together, it was still possible to perform the analysis. All the datasets passed the Shapiro–Wilk test for normality and were analyzed using a two-way ANOVA test. The comparison of the parameters identified in native vessel segments with parameters found in their decellularized counterparts revealed only one significant difference: Young’s modulus E_0_ of the proximal part of the native artery (CN, 0.128 ± 0.074 MPa) was significantly higher than E_0_ of the proximal part of the decellularized vessel (FD, 0.105 ± 0.068 MPa) (Figure 7C top left). This confirms that the decellularization procedure does not affect the mechanical properties even on the level of individual segments, and it is also in line with the findings from the whole artery analysis considering E_0_ and E_1_. The interesting finding of ultimate stress and strain increase in the case of CN and CD samples was not confirmed here, most probably due to a smaller dataset used for this individual analysis.

##### 3.2.2.3 Comparison of Arterial Segments Within the Groups

When comparing the different segments within the individual vessel groups, we observed significant differences between these segments in both fresh and cryoprotected native vessels (FN and CN). This confirmed the differences in porcine arteries that were reported earlier (Kochová et al., 2012). In particular, the mechanical parameters E_0_, E_1,_ and ultimate stress for the proximal segment have a clear trend to be lower than the values of middle and distal segments (apart from the ultimate strain). This trend was found to be statistically significant for E_0_ and E_1_ values of proximal (0.128 ± 0.074 MPa and 1.47 ± 0.55 MPa, respectively) and distal (0.851 ± 0.702 MPa and 6.5 ± 3.89 MPa, respectively) parts of CN vessels; for E_1_ values of proximal (1.54 ± 1.1 MPa), middle (6.48 ± 2.08 MPa), and distal (6.3 ± 2.46 MPa) parts of the FN vessels; and for the ultimate stress values of proximal (0.94 ± 0.43 MPa) middle (2.86 ± 1.42 MPa), and distal (2.97 ± 1.62 MPa) regions of the FN vessels. Moreover, we observed that these differences in native tissues can be transferred onto the decellularized scaffolds as in the case of Young’s modulus E_1_, where the value of the proximal part (3.08 ± 2.63 MPa) of the CD vessels was significantly lower than the E_1_ of its middle region (9.67 ± 4.61 MPa). The only exception was E_0_ in the FD group, where the mean value between the distal (0.125 ± 0.184 MPa) and proximal (0.105 ± 0.068 MPa) part was about the same.

Overall, the distal region presented higher values of E and ultimate stress and lower values of ultimate strain when compared to the proximal region due to lower elastin fiber content. If the elastin fibers are more abundant, the corresponding region of the artery, that is, the proximal part is more elastic and can reach a longer elongation. This is, in a way, supported by the ultimate strain values which were showing a trend of the proximal parts being higher than the other two parts, middle and distal (Figure 7C, bottom right). However, this trend was not proven to be significantly different. Young’s modulus E is the ratio between the stress and strain. If we hypothetically consider an equal stress but higher strain for these proximal parts, then we would expect the E values to be lower as a consequence. This is, indeed, shown in the graphs for both E_0_ and E_1_ values of the proximal parts, in some cases being statistically significant (Figure 7C, top left and right, respectively).

### 3.3 *In Vitro* Experiments

**FIGURE 8:**
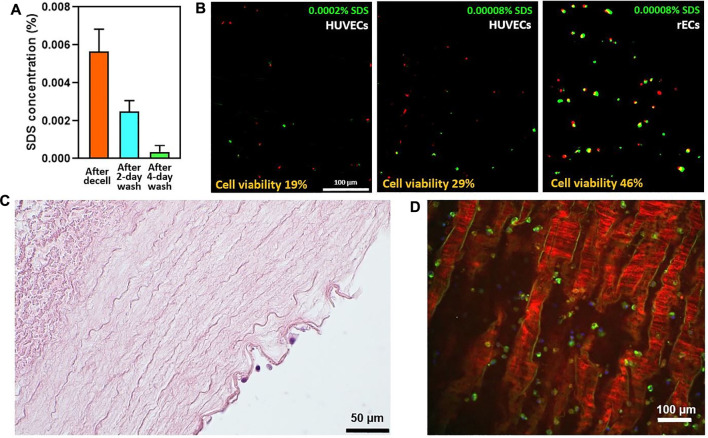
Endothelial cell culture in arterial scaffolds after 1 day: **(A)** SDS concentration with successive PBS washes; **(B)** Live/Dead assay images of three different 1-day seeding with HUVECs and rECs at different SDS concentration; live cells are depicted in green and dead cells in red; **(C)** Cells (dark blue) adhered to the ECM of tunica intima (H&E staining, vessel cross-section); **(D)** Vessel scaffold surface with attached cells (blue for nuclei; green for cytoskeleton) imaged by fluorescence microscopy; red fibers represent collagen I from the scaffold.

The compatibility of vessel scaffolds with the cells was analyzed by culturing scaffold stripes for 24 h with HUVECs. We also tested scaffold compatibility with rat ECs (rECs). As the concentration of residual detergents was still too high immediately after decellularization (scaffold storage solution showed mild foaming after a vigorous shake), we applied an additional 2-day wash and seeded the cells. At the same time, residual SDS concentration was measured both in solution after decellularization and in washing solution after a 2-day wash, with the SDS concentration decreasing from 0.0057% ± 0.0012–0.0025% ± 0.0006 (Figure 8A). Nevertheless, the cells did not show viability on the scaffolds even at this low SDS concentration (data not shown). Thus, we applied additional washing steps to obtain the 4-day washed scaffolds, with SDS concentrations reaching values very close to zero (the average value for all vessels evaluated was 0.0004% ± 0.0003, Figure 8A). With scaffolds prepared this way, we performed several 1-day *in vitro* experiments. The samples were assessed for cell viability and stained for cell and ECM protein presence by H&E and IF, respectively. The live/dead assay revealed that only some cells were kept alive even on 4-day washed scaffolds as the cell viability was 19% ± 12 and 29% ± 5 for HUVECs and 46% ± 15 for rECs representing three independent experiments (Figure 8B). The difference between the two HUVEC experiments seemed to be again linked to the residual SDS concentrations, which were still quite variable. 0.0002% of SDS was detected for scaffolds entering the experiment with 19% HUVEC viability, and 0.00008% was shown for experiments with 29% HUVECs and 46% rECs viability (Figure 8B, detailed values in Supplementary Table 3). The best viability was, thus, shown for rat endothelial cells on scaffolds with 0.00008% residual SDS that kept about half of the cells alive.

HUVEC culture on arterial scaffolds was also monitored by H&E staining and IF. Figure 8C shows an H&E image of a vessel cross-section with few HUVECs adhered. A perpendicular view of the vessel intima surface is shown in Figure 8D, where collagen I fibers were stained by immunofluorescence in red, and the cells are depicted in green. Sparse distribution and partially rounded cell shape are likely related to residual SDS content which was around 0.0002% in this experiment.

## 4 Discussion

The need for small-caliber vessel grafts and the problems of thrombogenicity related to the use of polymeric materials have led to the development of acellular xenogeneic grafts (Fayon et al., 2021). Protocols for decellularization of carotid arteries from different species have been developed; however, they last for more than 1 day, making it difficult to adapt these lengthy procedures for future clinical use where timing is essential (Dahl et al., 2003; Roy et al., 2005; Dahan et al., 2012; Sheridan et al., 2012; López-Ruiz et al., 2017; Wang et al., 2017; Gu et al., 2018; Cai et al., 2019; Cheng et al., 2019; Cai et al., 2020). Thus, we decided to contribute to this field by developing a procedure that would be faster than the ones currently used, while maintaining the scaffold well-preserved.

### 4.1 High Tissue Protection

First , we focused on careful tissue explantation to maintain the vessels as intact as possible for future processing. This was facilitated by two factors: 1) having a large animal house available just next to the laboratory, and 2) long-term collaboration with the team of surgeons working in the neighboring faculty hospital who performed the explantation.

We also compared scaffolds that were freshly decellularized with vessels that were stored for a period of time at −80°C in a cryoprotective solution to increase the immediate accessibility of tissues for the future. The decellularization of a fresh artery without any unnecessary delay has the advantage to obtain a scaffold that is the closest to the native ECM. Generally, prolonged cold storage of vessels prior to decellularization, for example, during transportation might result in the damage of the ECM proteins. The cryopreservation, on the other hand, would keep the vessel stored until it is needed. Our approach to cryoprotection might seem contradictory to many decellularization protocols that suggest using freeze/thaw cycles for an increase in decellularization efficacy (Porzionato et al., 2018; Cheng et al., 2019). Plain freezing and thawing can, indeed, help cell removal. However, if complete decellularization can be achieved without this step, the natural structure of the ECM can be better maintained as possible protein damage caused by enzymes released from cells during uncontrolled freezing and thawing can be prevented.

We considered the contact time of the tissue with the detergent to be of great importance. Recently, we developed a fast protocol for decellularization of porcine liver lasting around 3 h (Moulisová et al., 2020), where we particularly focused on shortening the period of SDS perfusion. SDS is known for being very efficient but is also a harsh detergent, and thus some protocols for carotid arteries use a low SDS concentration (Cai et al., 2019; 2020) or completely avoid this reagent (Neff et al., 2011; Dahan et al., 2012; Sheridan et al., 2012). Nevertheless, using lower SDS concentrations or less efficient detergents results in much longer decellularization protocols, extending the time of arterial scaffold production, which takes 3 days and more (Neff et al., 2011; Dahan et al., 2012; Sheridan et al., 2012; Cai et al., 2019). Our approach is based rather on the strategy of shorter tissue contact with detergents as it seems to be better for the preservation of the scaffold’s inner structure and provides the scaffold in shorter time.

### 4.2 Decellularization Efficiency

However, the efficiency of our fast protocol adapted from vein decellularization appeared to be lower for arteries. In the case of veins, all cells were always removed (Massaro et al., 2021a), while only a proportion of the arteries was completely decellularized. Naturally, when compared to the veins, the arterial vessel wall is much thicker and more compact due to the presence of elastic layers and much higher abundance of smooth muscle cells. Still, some of the arteries were decellularized completely, which could be related to the variable distribution of elastic fibers within the vascular wall as described by Espinosa et al. (2018). Our more detailed analysis revealed higher efficiency for arteries decellularized from fresh tissue. This can be possibly explained by mild tissue damage, particularly of endothelial cells caused by cold ischemia during the typical overnight storage period of these vessels prior to decellularization. This damage is considered minor in the case of vessel graft storage prior to implantation for up to 24 h, however depending strictly on the preservation solution (Neil et al., 2002). According to that study, saline (which we used as our storage solution in this study) did not produce such strong damage related to mitochondria, cytoplasm, and nuclear structure in endothelial cells of rat aortas (Neil et al., 2002). Still, in our case, this mild damage could have an effect on easier accessibility of detergents into cells than that of the cells of vessels that were cryopreserved immediately after explantation and fast-thawed to avoid cell damage by ice crystal formation (Amini et al., 2020). Moreover, we observed high variability in the content of elastic fibers between individual animals, which can also contribute to the different levels of decellularization, disregarding the storage conditions. The role of interindividual variability is also supported by a recent report describing a clear effect of heterogeneity between individuals on the decellularization outcome in the case of human cardiac tissue and related limitations of standardized protocols (Tenreiro et al., 2021).

### 4.3 Good Protein Maintenance

We put in a substantial effort on evaluating the quality of the scaffold with respect to the molecular composition. We also considered the relationship between the vascular layer, the segment of the vessel on the one hand, and the mechanical strength on the other hand.

All three different layers of the arterial wall (tunica intima, media, and adventitia, as shown in Figure 2A) were visualized by several microscopic techniques. Based on the evaluation of microscopic images of samples, both native and decellularized, we assume the protein composition of the ECM of these three layers does not change after the decellularization procedure. Cai et al. described a decrease in the biochemical properties of porcine carotid arteries after using 1% of Triton X-100 and 0.3% SDS solutions in a 4-day long decellularization protocol (lower collagen content and looser scaffold structure) (Cai et al., 2019). Our study, on the other hand, confirmed good maintenance of ECM proteins of high importance such as collagen, elastin, fibronectin, and laminin using a combination of classical histological staining such as H&E, Picrosirius red, Verhoeff’s green trichrome, and orcein with specific immunofluorescence stainings. This implies that reasonable optimization of the protocol (increased SDS concentration, shortening the incubation time) can result in a high-quality scaffold to be used for cell repopulation. Other studies report that the protein content unchanged after the decellularization; however, the time to obtain these scaffolds is still longer than the one we report here (Sheridan et al., 2014; Cheng et al., 2019; Ilanlou et al., 2019).

### 4.4 Mild GAG Loss

Regarding the importance of GAGs for cytokine and growth factor retention in vessels [reviewed in (Lepedda et al., 2021)], we also included GAG staining in our study. We showed that native arteries have a high content in tunica media, while, basically, no GAGs are present in the adventitia. Based on histological staining, our decellularization protocol leads to a decrease in GAG content in tunica media, but still, some GAGs were preserved. A similar decrease has also been reported by other groups studying porcine carotid arteries (Cai et al., 2019; Cheng et al., 2019), while other groups did not monitor GAG presence at all (Sheridan et al., 2012). There are studies reporting that GAG loss is directly related to the SDS effect (Uygun et al., 2010) or to the effect of Triton X-100 (Mendoza-Novelo et al., 2011) decellularizing rat liver and bovine pericardium, respectively. However, considering the lower effectivity and prolonged decellularization procedure when other decellularization agents are used (Grandi et al., 2011; Kajbafzadeh et al., 2017), we assume the mild loss of GAGs could be an acceptable compromise when obtaining the scaffold faster and of high quality in other aspects such as protein content and mechanical strength. Nevertheless, whether this GAG decrease affects the repopulation procedure has to be investigated.

### 4.5 Retention of Mechanical Properties

Tunica media comprises mainly of smooth muscle cells and fibers of elastin and collagen. The decellularized ECM retains these structurally important fibers; thus, tunica media is the vessel part mainly responsible for the mechanical strength of the vessel. The external layer of the arterial wall, the adventitia, comprises fibroblasts and collagen fibers. Its role is to provide stability to the wall, and its presence depends on the type of artery (Holzapfel et al., 2000). Importantly, carotid arteries have been shown to vary between proximal and distal parts in the percentage of collagen, elastin, and actin (Tomášek et al., 2020). The elastin content decreases in favor of the smooth muscle cells and collagen content from the proximal toward the distal part of the vessel wall and inevitably influences the mechanical properties of different vessel segments (Tomášek et al., 2020). This suggests that the mechanical properties of different parts of decellularized scaffolds can also be influenced. Our detailed analysis of each individual segment confirmed that proximal vessel segments showed a higher ultimate strain and thus lower stiffness (Young’s modulus) than middle and distal segments that were also supported by some data from decellularized scaffolds. Thus, the segmentation of the scaffolds should be taken into account in future processing to ensure that a suitable segment is used, and random cutting of porcine carotid arteries in segments for the decellularization (Sheridan et al., 2012) should be avoided.

Previous studies reported weakened mechanical properties of porcine carotid artery scaffolds in comparison to native vessels and suggested different types of crosslinking to overcome this obstacle (Gu et al., 2018; Cai et al., 2019). Our work shows it is possible to obtain completely decellularized vessel scaffolds using a shorter protocol with mechanical properties maintained; thus, no additional complex modifications to restore the compromised quality are needed.

From the mechanical study, we only observed a difference between native and decellularized tissue in the case of opening angle values. The opening angle indicates the presence of residual stresses in the arterial wall that are mainly attributed to the smooth muscle cells (Rachev and Hayashi 1999). Thus, the loss of smooth muscle cells results in significant reduction of the circumferential stress. The results from both analyses and comparisons of opening angles of the whole arteries and individual segments are well-corresponding with this theoretical decrease of residual stress. Considering individual values, our results (56° for the native groups and 25° for the decellularized groups) are in line with those of Roy et al. that obtained opening angles around 69° in the native and 26° in the decellularized samples of porcine carotids (Roy et al., 2005).

Given that a decellularized vessel loses the cells in the process, we need to quantify if this loss influences its behavior. In order to increase our knowledge further, we decided to consider not only the differences between the native tissue, scaffold, and the single main regions of the artery but also include different storing conditions. We believe that for future translational use, the possibility to keep the native tissue and/or the scaffold stored would be beneficial in terms of the availability of the graft. Our findings evidenced that the decellularization procedure did not have an impact on the mechanical behavior in the longitudinal direction.

Looking at the vessels from the direction that is perpendicular to the longitudinal, Holzapfel et al. showed the influence of each layer of the arterial wall on the mechanical behavior of human coronary arteries (Holzapfel et al., 2005). They found out, by separating each layer, that the tunica adventitia can reach a stress of 1.3 MPa until rupture, while the tunica media and intima can only arrive at 0.4 MPa. Although we did not investigate the mechanical properties of each arterial layer separately in this study, we conclude it is important to keep all layers together for obtaining the scaffold which is as close to the native vessel as possible. Considering this aspect, we can state that our decellularized arteries present similar values as the native ones.

### 4.6 Compatibility With Cells

Tunica intima, the inner layer, is formed by endothelial cells to create a barrier between blood and the vessel wall. The endothelium is, thus, responsible for the antithrombogenicity of native blood vessels. There are studies successfully using endothelial cells for vessel scaffold repopulation to restore the intima (McFetridge et al., 2004; Quint et al., 2011; Dahan et al., 2012). It is important that the matrix stimulates cell adhesion and growth to reduce the risks of rejection and graft failure. In this work, we included a scaffold cytocompatibility experiment with two different cell types to test porcine scaffold compatibility with endothelial cells. We showed that both HUVECs and rECs can survive on the scaffold; nevertheless, we clearly showed that significant removal of residual detergents is absolutely necessary to keep the endothelial cells alive during repopulation. Our findings related to HUVEC sensitivity to residual SDS concentrations are in line with the publication of Zvarova et al. describing a very high susceptibility of endothelial cells, including HUVECs to low concentrations of this detergent (Zvarova et al., 2016). They report that endothelial cell types are affected by SDS concentrations as low as 0.00012%; at the same time, they show that other cell types are not so vulnerable, for example, hMSCs tolerate values around 0.002%. Apart from HUVECs, we also tested rat endothelial cells which were shown to be less susceptible than their human counterparts. Our results showing the high variation between cell viability of HUVECs and rECs on a matrix with comparable SDS residual content newly suggests different SDS sensitivity of endothelial cells from different species which has not yet been reported. At the same time, our findings highlight the importance of SDS residue monitoring in decellularized matrices. Provided that the SDS detergent residues are carefully removed from the scaffold, the cells would eventually be able to completely repopulate the scaffold and remodel the ECM for complete integration in the long term (Dahan et al., 2012).

## 5 Conclusion and Future Plans

Our study showed that using our fast protocol, we could produce fully decellularized porcine carotid arteries with well-preserved ECM components and retain the mechanical characteristics of the native vessels. Although this protocol was not 100% efficient, it still brought the benefit of obtaining high-quality scaffolds. Moreover, we introduced a novel strategy of vessel cryopreservation using cryoprotective solution prior to the decellularization, which brings not only the benefit of the vessel available on demand but also better protection of the arterial ECM structure. We also confirmed the results of a previous study on the difference in mechanical properties in individual arterial segments and showed that this could be transferred onto the decellularized scaffolds. Finally, the thorough removal of SDS residues from scaffolds was shown to be crucial for endothelial cell survival after seeding. The next steps we are planning in this area are to modify the protocol to increase the decellularization efficiency and perform a detailed study on the scaffold repopulation with primary cells with *in vivo* testing as the proof of concept. In parallel, we would like to expand the mechanical testing toward the analysis of the inflation-extension tests. As the arteries have a physiological pressure range in the circumferential direction corresponding to systolic and diastolic pressures, the knowledge of the maintenance of such mechanical properties in scaffolds and eventually recellularized grafts is crucial when the risk of aneurysm development is considered.

## Data Availability

The original contributions presented in the study are included in the article/Supplementary Material, further inquiries can be directed to the corresponding author.
