# Supplementary material for: Decellularization of Porcine Carotid Arteries: From the Vessel to the High-Quality Scaffold in Five Hours
Source: Front Bioeng Biotechnol. 2022 May 16;10:833244. doi: 10.3389/fbioe.2022.833244 (PMC9150822; doi:10.3389/fbioe.2022.833244)

Supplementary Material

1 Supplementary Figures and Tables, Videos

1.1 Supplementary Figures

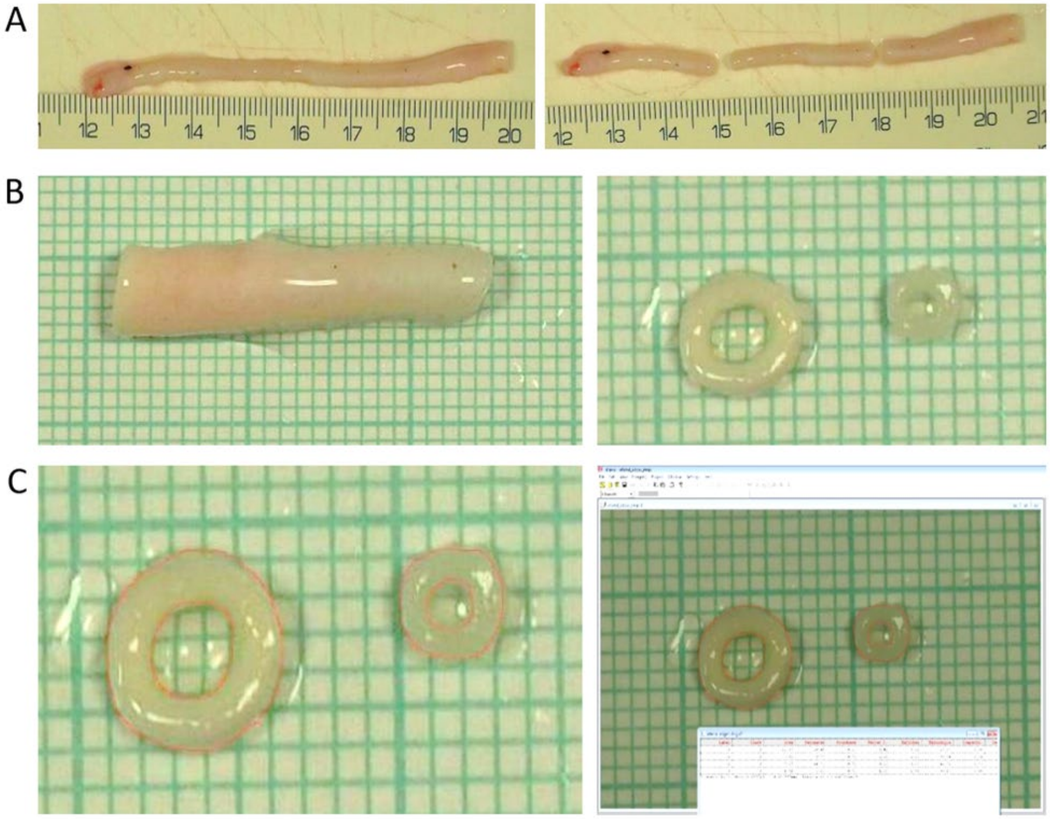

Supplementary Figure 1. Mechanical testing – documentation of samples and measurements: **(A)** Larger parts of vessels next to the ruler (left – whole vessel, right – the same vessel cut in three segments); **(B)** smaller vessel parts on a graph paper (left – arterial segment prior mounting into the testing device, right – rings cut off both ends of the segment), **(C)** image processing in Ellipse software (left – manual annotation of the rings, right – print screen of Ellipse software with calculated values for annotated rings). Graph paper scale: small square is equivalent to 1 x 1 mm.

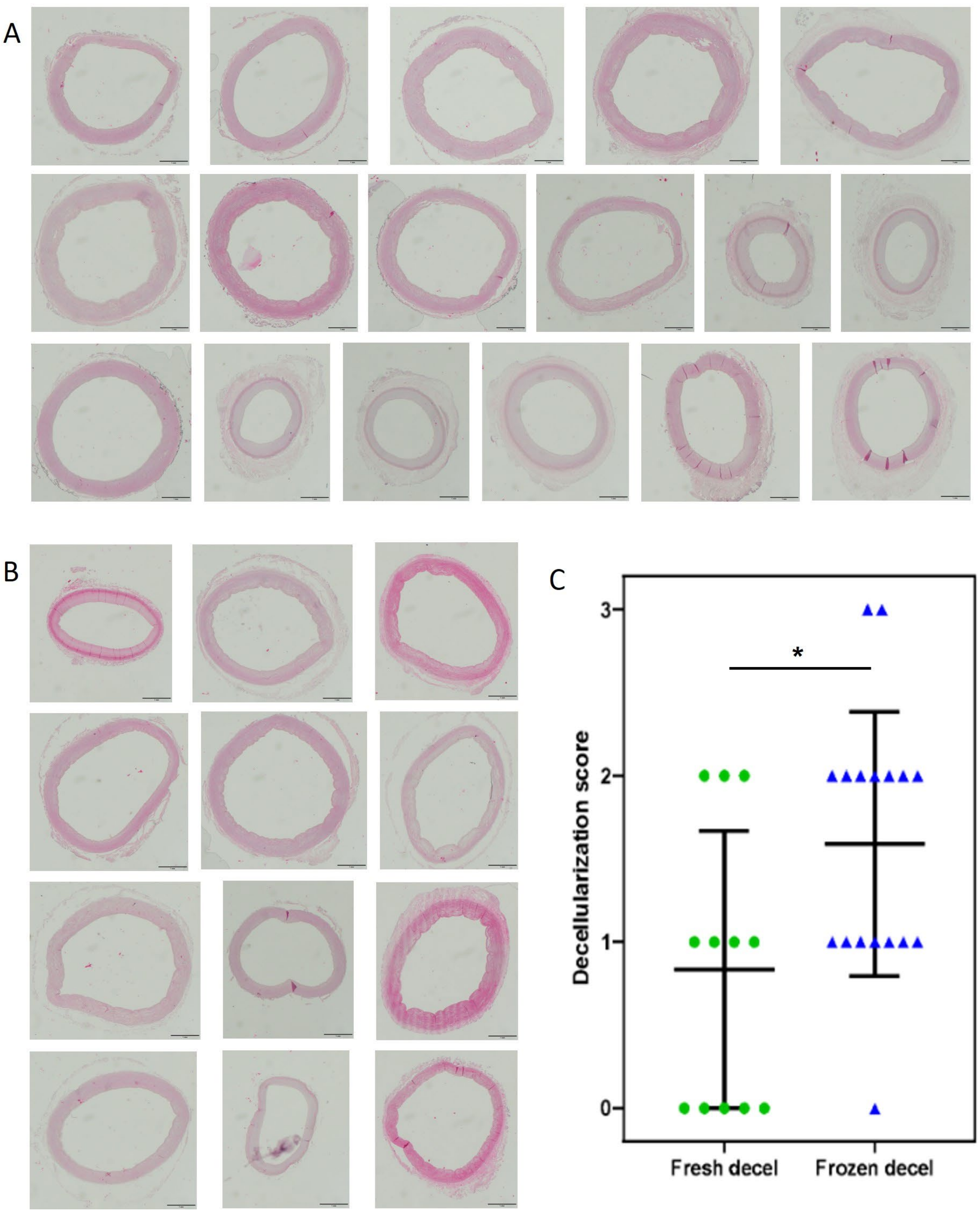

Supplementary Figure 2. (A) H&E image of the decellularized scaffolds that were cryopreserved prior decellularization (CD), **(B)** H&E image of the decellularized scaffold treated within 24 hours from the harvest (freshly decellularized, FD), **(C)** boxplot of the scoring system to assess the decellularization level. From the boxplot we can see that the decellularization is more efficient when conducted on freshly explanted vessels.

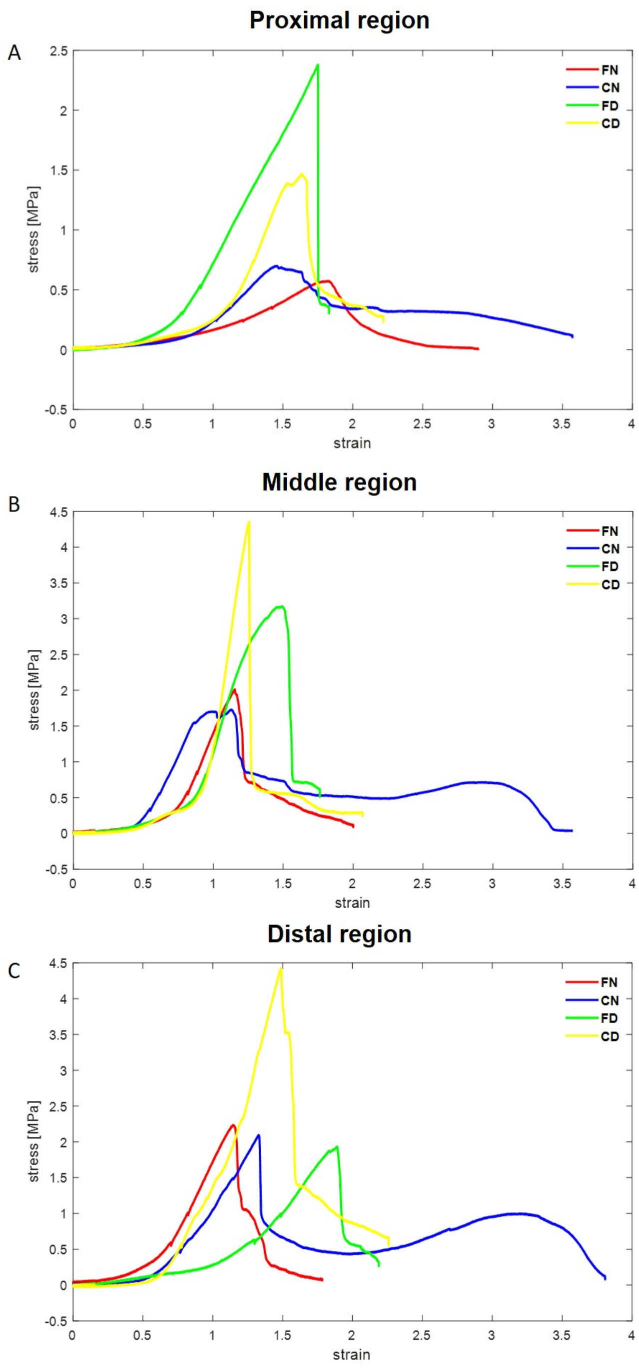

Supplementary Figure 3. Stress-strain curves of one representative sample per each of the four groups analyzed, divided between proximal **(A)**, middle **(B)** and distal **(C)** regions. The behavior of each segment is equal, with an elastic initial region and a viscoelastic region before rupture. The variation is on the values reached by the decellularized scaffolds in terms of ultimate stress and/or ultimate strain when compared to the native carotid artery.

1.2 Supplementary Tables

Supplementary Table 1: Scoring criteria for the evaluation of the decellularization efficiency

**Score number**	**Description**
0	whole scan is completely nuclei-free
1	few nuclei remaining, usually in the central part of the vessel (less than 20% of the tissue area)
2	some nuclei left in different parts of the tissue section (on 20%- 50% of tissue area)
3	many nuclei left (more than 50% of the tissue area)

Supplementary Table 2: Mechanical properties of native carotid arteries and decellularized scaffolds calculated for individual segments as well as averaged for the whole vessel; detailed parameter values (mean value with standard deviation) for opening angle, Young moduli E_0 and E_1 and ultimate stress and strain. Vessels used: Fresh native (FN, n=10), cryopreserved native (CN, n=8), decellularized from fresh tissue (fresh decellularized, FD, n=8) and decellularized from frozen tissue (cryopreserved decellularized, CD, n=6).

		**FN**	**CN**	**FD**	**CD**
**Opening angle**	Distal	65°±19	46°±19	26°±5	20°±12
	Middle	61°±17	44°±24	25°±23	15°±17
	Proximal	55°±12	66°±22	12°±16	37°±18
	All segments	60°±16	52°±23	22°±18	24°±18
**E_0 [MPa]**	Distal	0.504±0.426	0.851±0.702	0.125±0.184	0.306±0.155
	Middle	0.565±0.514	0.469±0.466	0.264±0.195	0.306±0.062
	Proximal	0.141±0.094	0.128±0.074	0.105±0.068	0.182±0.263
	All segments	0.403±0.421	0.483±0.556	0.177±0.181	0.264±0.179
**E_1 [MPa]**	Distal	6.3±2.46	6.5±3.89	3.69±1.54	5.52±2.69
	Middle	6.48±2.08	5.38±3.15	7.12±5.24	9.67±4.61
	Proximal	1.54±1.1	1.47±0.55	2.05±0.75	3.08±2.63
	All segments	4.77±3	4.45±3.55	4.73±3.93	6.09±4.27
**Ultimate stress [MPa]**	Distal	2.97±1.62	1.76±1.09	1.43±0.64	2.31±1.19
	Middle	2.86±1.42	1.66±1.01	2.5±1.34	3.40±1.19
	Proximal	0.94±0.43	0.71±0.3	1.52±0.69	1.81±1.68
	All segments	2.26±1.55	1.38±0.96	1.88±1.08	2.51±1.46
**Ultimate strain**	Distal	0.79±0.3	0.45±0.18	0.86±0.46	0.96±0.12
	Middle	0.85±0.49	0.55±0.24	0.74±0.29	0.79±0.2
	Proximal	0.97±0.4	0.86±0.37	1.22±0.16	1.37±0.43
	All segments	0.87±0.4	0.62±0.32	0.88±0.38	1.04±0.37

Supplementary Table 3: Scaffold SDS residue concentrations mesured either in storage solution the scaffold was kept after decellularization or in washing solutions after applying washing steps.

Experiment	Replicate 1 (%)	Replicate 2 (%)	AVG (%) single experiment	AVG (%) all experiments	SD all experiments
After decell/1	0.00666	0.00609	0.00638		
After decell/2	0.00659	0.00722	0.00691		
After decell/3	0.00421	0.00393	0.00407		
After decell/4	0.00519	0.00526	0.00523	**0.00565**	**0.00119**
After 2-day wash/1	0.00307	0.00318	0.00313		
After 2-day wash/2	0.00281	0.00288	0.00284		
After 2-day wash/3	0.00174	0.00174	0.00174		
After 2-day wash/4	0.00248	0.00206	0.00227	**0.00249**	**0.00058**
After 4-day wash/1	0.00027	0.00016	0.00022		
After 4-day wash/2	0.00027	0.00022	0.00025		
After 4-day wash/3	0.00092	0.00085	0.00088		
After 4-day wash/4	0.00016	0.00000	0.00008	**0.00036**	**0.00034**

1.3. Videos

Video 1: Running decellularization procedure of two porcine carotid arteries; the first washing step before perfusion with Triton X-100.

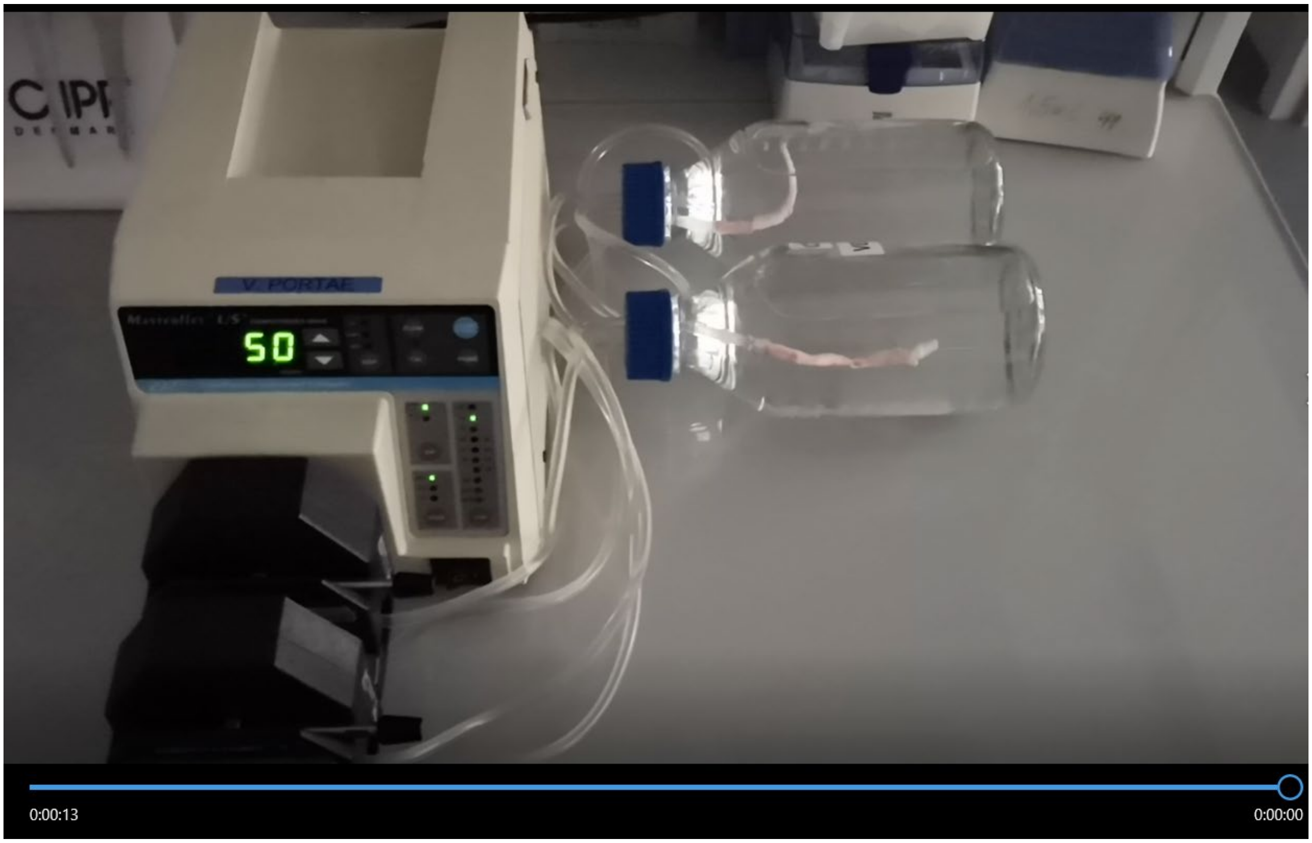

Supplement: Supplementary file 1 [file DataSheet1.pdf]
